# The effect of different dose of heparin using in peripheral arteriovenous synchronous blood exchange transfusion for neonatal hyperbilirubinemia

**DOI:** 10.5937/jomb0-45223

**Published:** 2024-01-25

**Authors:** Xueyun Guan, Jing Guo, Dongsu Xiao, Zhong Wu

**Affiliations:** 1 Ganzhou People's Hospital, Department of Pediatric, Ganzhou, China; 2 Ganzhou People's Hospital, Department of Gastrointestinal Surgery, Ganzhou, China

**Keywords:** heparin, hyperbilirubinemia, neonatal, peripheral arteriovenous synchronous blood exchange transfusion, heparin, hiperbilirubinemija, neonatalna, periferna arteriovenska sinhrona transfuzija krvi

## Abstract

**Background:**

To explore the optimal dosage of heparin in peripheral arteriovenous automatic synchronous exchange transfusion therapy for neonatal hyperbilirubinemia.

**Methods:**

A total of 185 neonates received peripheral arteriovenous synchronous blood exchange transfusion for hyperbilirubinemia were enrolled from pediatric department of the Ganzhou People's Hospital between January 2018 and June 2020, which were randomly divided into four groups. On the basis of exchange transfusion, different dose of heparin was pumping at the bleeding site of artery (A: no heparin; B: 100 U/h heparin; C: 200 U/h heparin; D: 300 U/h heparin). The indexes of exchange transfusion efficacy, including total bilirubin conversion rate, indirect bilirubin conversion rate, hemoglobin concentration, the platelet number and APTT value was measured before and after therapy. The sites of artery puncture, the sites and rate of vascular occlusion were counted and analyzed.

## Abstract (sr)

**Uvod:**

Cilj je bio da se istraži optimalna doza heparina u perifernoj arteriovenskoj automatskoj sinhronoj transfuzijskoj terapiji za neonatalnu hiperbilirubinemiju.

**Metode:**

Ukupno 185 novorođenčadi primilo je perifernu arteriovensku sinhronu transfuziju krvi zbog hiperbilirubinemije sa pedijatrijskog odeljenja Narodne bolnice u Ganzhou između januara 2018. i juna 2020. godine, koji su nasumično podeljeni u četiri grupe. Na osnovu izmene transfuzije, različite doze heparina su pumpane na mestu krvarenja arterije (A: nema heparina; B: 100 U/h heparina; C: 200 U/h heparina; D: 300 U/h heparina). Indeksi efikasnosti razmene transfuzije, uključuju i stopu konverzije ukupnog bilirubina, stopu konverzije indirektnog bilirubina, koncentraciju hemoglobina, broj trombocita i vrednost APTT mereni su pre i posle terapije. Mesta punkcije arterija, mesta i stopa vaskularne okluzije su prebrojani i analizirani.

**Rezultati:**

Nije bilo značajne razlike u stopi konverzije ukupnog bilirubina, stopi indirektne konverzije bilirubina, koncentraciji hemoglobina u broju trombocita i vrednosti APTT pre i posle transfuzije izmene između četiri grupe (P >0,05). Pored toga, nije bilo statističke značajnosti u mestima punkcije arterija između četiri grupe (P > 0,05). Utvrđena je značajna razlika u ukupnoj stopi vaskularne okluzije i stopi okluzije radijalne arterije između četiri grupe (P<0,05).

**Zaključak:**

Različite doze heparina koje se koriste u perifernoj arteriovenskoj sinhronoj transfuziji krvi nisu uticale na terapijski efekat ili koagulacionu funkciju pacijenata, ali su mogle da smanje stopu okluzije u zavisnosti od mesta arterijske punkcije.

## Introduction

Hyperbilirubinemia, characterized by the high total serum bilirubin level, is the most common disorder in neonates [1]. It is reported that about 60% of infants would appear clinically condition in the first week of life [2]. Severe hyperbilirubinemia may lead to various complications, including lifelong disability and neurodevelopmental impairment [3]. Although timely detection and effectively intervention and treatment can help prevent the bilirubin-induced sequelae, management of severe hyperbilirubinemia remains a challenge.

Exchange transfusion (ET) is the removal of the infant's blood and simultaneous replacement with fresh donor blood. ET could rapidly reduce the bilirubin level, so it is an effective treatment for severe hyperbilirubinemia when intensive phototherapy has no effect or when hemolysis is excessive [4] [5]. However, as an invasive procedure, ET is usually associated with adverse events including sepsis, vascular accidents, cardiovascular compromise, and air embolism [6] [7]. The factors including narrow diameter of the neonatal peripheral artery and the uncooperating of infant make the puncture difficult. Moreover, the whole ET process is often interrupted due to arterial occlusion, which prolongating the time and increasing the risk of ET [8]. Thus, keeping arteries free of obstructions is one of the key points and difficulties in the process of ET. 

Heparin, a highly sulfated polysaccharide, which plays functional roles in many kinds of biological activities [9]. It was reported that heparin could inhibited blood coagulation cascade to keep blood flowing normal in the vasculature [10] [11]. At present, heparin is mainly used to avoid uncontrolled thrombosis and maintain arterial patency during in the process of exchange transfusion [12] [13]. However, the usage and dosage of heparin are different in application. The dosage of heparin is mostly between 30 U H-300 U/h, but heparin-free blood exchange transfusion therapy was also used [14]. The tube blocking rate will increase when the dose of heparin is too small, the coagulation function of the body will be impaired when the dose of heparin is too high. Therefore, it is particularly important to choose the most appropriate dose of heparin in clinic. The aim of this study was to explore the optimal dosage of heparin in peripheral arteriovenous synchronous blood exchange transfusion therapy for neonatal hyperbilirubinemia.

## Materials and methods

### General data

The newborns who received peripheral arteriovenous synchronous blood exchange transfusion for hyperbilirubinemia were enrolled from pediatric department of the Ganzhou People's Hospital between January 2018 and June 2020. Inclusion criteria including: (1) Patients were diagnosed with neonatal hyperbilirubinemia [15]; (2) The guardians of the patients were aware of the study and signed the informed consent for exchange transfusion and heparin treatment. Patients with one of the following diseases were excluded: (1) Patients were found with abnormal coagulation function before exchange transfusion; (2) Difficulty in arterial puncture (arterial puncture was performed more than 3 times); (3) Exchange transfusion was aborted due to changes in patients' conditions. A total of 185 cases of hyperbilirubinemia were enrolled in this research. There were 106 males and 79 females with a gestational age of 37.8 ± 1.9 weeks and a birth weight of 3128.0 ± 792.0 g. The diseases including ABO hemolytic disease (89 cases), rhesus incompatibility hemolytic disease (36 cases), G-6-PD deficiency (31 cases), septicemia (20 cases) and others (9 cases). Patients were randomly divided into four groups: A group without heparin treatment (45 cases); B group with 100 U/h heparin treatment (47 cases); C group 200 U/h heparin treatment (47 cases); D group with 300 U/h heparin treatment (46 cases). All procedures were approved by the Ethics Commitment of Ganzhou People's Hospital.

### Interventions 

(1) Blood type selecting and ration: In the case of Rh incompatibility, ABO compatible, RhD negative RBCs were used. In the case of ABO incompatibility, group O red blood cell were reconstituted with group AB plasma. Use fresh blood collected within 3 days, and the volume of exchange transfusion was prepared as twice the blood volume of the neonates (150-180 mL/kg). The blood is heated to 36~37 using a heating instrument. (2) Preoperative preparation: Stop feeding the baby once before blood exchange. Half an hour before surgery, patients were sedated with intravenous use of phenobarbital. If the children were agitated during surgery, chloral hydrate was administered through enema injection. The newborns were placed on the radiation table, wrapped in Bird's Nest, and comforted with pacifier. The skin temperature of newborns was maintained at 36.3 ∼ 37.3°C, and the bleeding limb was fixed in the bird's nest with bandages. (3) Monitoring of vital signs: Multi-function ECG monitor was used to continuously monitor the heart rate, respiration rate and percutaneous oxygen saturation of patients. The blood pressure was measured every 15 minutes. The patients' skin color, muscle tension and mental reaction were observed all the time. 

(4) Establishment of arteriovenous passageway: Three peripheral venous channels were established, two of the channels were connected to blood bags through blood transfusion devices for plasma infusion and red blood cell washing, another channel used for medication during blood exchange transfusion. The blood transfusion speed is controlled by the blood transfusion pump, which is the port of blood transfusion. One peripheral arterial vascular channel was established through 24GY type of indwelling needle. In no heparin group, the arterial catheter was directly connected with the discharge channel. In other three groups, the arterial catheter was connected with a three-way tube, and the other end was connected with the discharge channel. The whole blood exchange route was closed.

(5) Fluid velocity: At the beginning, the speed of blood transfusion was slow. After 10 minutes, if the vital signs of patients were normal, the speed could be improved under a doctor's supervision. Exsanguination speed = transfusion speed + heparin saline injection speed. The whole exchange transfusion process lasted for 2-2.5 hours. (6) Blood analysis: Blood samples were collected before, in the process (the volume of blood exchange reaches half of the total volume) and after exchange transfusion.

### Laboratory examination

The blood biochemistry, blood routine, coagulation function as well as blood gas analysis were examined.

Effect of exchange transfusion: The bilirubin and hemoglobin levels were measured before and after exchange transfusion [16]. The exchange rate of bilirubin was calculated: Bilirubin exchange rate = (pre-exchange bilirubin value - post-exchange bilirubin value)/pre-exchange bilirubin value × 100%.

Artery Occlusion: The incidence and location of artery occlusion were observed and counted.

Coagulation function: The platelet number and APTT value were measured before and after exchange transfusion [17].

### Statistical analysis

The data were analyzed by IBM Statistic Package for Social Science (SPSS) 21.0 (Armonk, NY, USA), measurement data were presented as »mean ± SD«. Enumeration data were presented as frequency and percentage. Analysis of variance was used for the comparison of measurement data between multiple groups. Enumeration data were analyzed by chisquare test. P < 0.05 was considered statistically significant.

## Results

### The curative effect of different heparin dose on exchange transfusion

**Figure 1:**
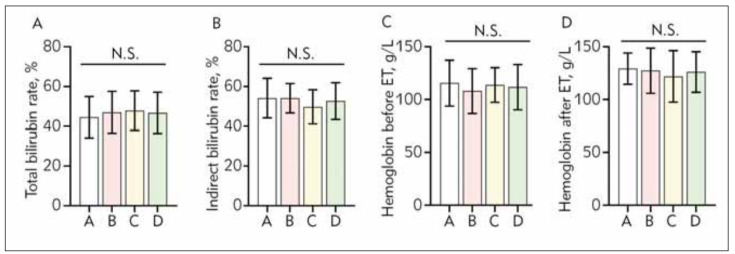
The results of measure analysis of variance for comparison of total bilirubin conversion rate, indirect bilirubin conversion rate, hemoglobin concentration before ET and hemoglobin concentration after ET. There was no between the four groups (P>0.05). Measurements are expressed as mean ± standard deviation. ET: exchange transfusion; A: no heparin group; B 100 U/h heparin group; C: 200 U/h heparin group; D: 300 U/h heparin group.

There was no significant difference for total bilirubin conversion rate, indirect bilirubin conversion rate, hemoglobin concentration before exchange transfusion and hemoglobin concentration after exchange transfusion between the four groups (P >0.05, Table 1) (Figure 1). In addition, there was no statistical significance in artery puncture point between the four groups (P > 0.05, Table 2).

**Table 1:** Comparison of bilirubin conversion rate and hemoglobin concentration between four groups

	A (n=45)	B (n=47)	C (n=47)	D (n=46)	F/χ^2^	P
Total bilirubin rate (%)	44.48±10.51	46.98±10.59	47.82±10.01	46.68±10.47	0.925	0.43
Indirect bilirubin rate (%)	54.19±9.95	54.12±7.41	49.76±8.60	52.69±9.21	2.42	0.068
Hemoglobin before ET (g/L)	115.54±21.78	108.04±21.10	113.79±16.35	111.76±21.41	1.213	0.306
Hemoglobin after ET (g/L)	129.36±14.75	127.30±21.36	121.93±24.43	126.09±19.19	1.101	0.35

**Table 2:** Comparison of artery puncture sites between four groups.

	A (n=45)	B (n=47)	C (n=47)	D (n=46)	F/χ^2^	p
Radial artery	29	34	30	31	1.819	0.936
Brachial artery	10	9	13	10		
Axillary artery	6	4	4	5		

### The rate of arterial occlusion

Significant difference in total rate of vascular occlusion was found between the four groups (P< 0.05), which were 15.6%, 31.9%, 21.3% and 8.7%, respectively. Further comparison showed that the rate of vascular occlusion of group A was significantly lower than that of groups B and C (P< 0.05); There was no significant difference in the total rate between the group A and group D (P > 0.05); Group D showed a significantly smaller total rate than groups B and C (P< 0.05); The total rate in group C was slightly lower than that in group B, but the difference was not statistically significant (P > 0.05). A significant difference in the rate of radial artery occlusion was found among the four groups (P < 0.05), the rate in group B were highest (31.9%), while that in group D was the lowest (6.5%). However, there was no significant difference in the rate of brachial artery occlusion and axillary artery occlusion among the four groups (P > 0.05, Table 3).

**Table 3:** Comparison of rate of arterial occlusion between four groups.

	A (n=45)	B (n=47)	C (n=47)	D (n=46)	F/χ^2^	p
Radial artery	6 (13.3%)	15 (31.9%) *	8 (17.0%)	3 (6.5%) *	11.251	0.01
Brachial artery	1 (22.2%)	5 (10.6%)	1 (2.1%)	1 (2.1%)	5.431	0.143
Axillary artery	0 (0%)	0 (0%)	1 (2.1%)	0 (0%)	2.756	0.431
** Total **	** 7 (15.6%) **	15 (31.9%) *	10 (21.3%) *	** 4 (8.7%) **	** 8.589 **	** 0.035 **

### The curative effect of different heparin dose on blood coagulation

**Figure 2:**
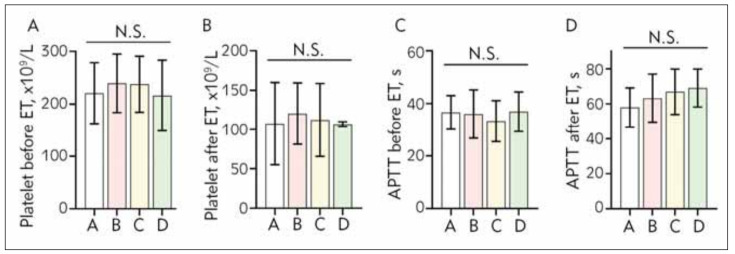
The results of measure analysis of variance for comparison of platelet number before ET, platelet number after ET, APTT value before ET, APTT value after ET. There was no between the four groups (P >0.05). Measurements are expressed as mean ± standard deviation. ET: exchange transfusion; A: no heparin group; B 100 U/h heparin group; C: 200 U/h heparin group; D: 300 U/h heparin group.

The platelet number and APTT value showed no significantly difference between four groups before and after exchange transfusion (P > 0.05, Table 4) (Figure 2).

**Table 4:** Comparison of platelet number and APTT value between four groups.

	A (n=45)	B (n=47)	C (n=47)	D (n=46)	F/χ^2^	P
Platelet before ET (×10^9^/L)	220.45±57.86	239.26±55.72	237.49±53.14	215.96±67.04	1.88	0.135
Platelet after ET (×10^9^/L)	107.58±52.40	120.48±38.92	112.38±46.23	107.10±3.20	0.923	0.431
APTT before ET (s)	36.57±6.39	35.98±9.21	33.29±7.83	36.87±7.52	2.235	0.086
APTT after ET (s)	57.88±11.16	63.22±13.70	66.84±12.92	68.95±10.80	2.274	0.082

## Discussion

Hyperbilirubinemia is one of the common diseases in neonates [18]. Severe hyperbilirubinemia with deposition of bilirubin in the central nervous system may lead to neurological dysfunction and even death [19]. Exchange transfusion therapy is the most rapid and effective treatment for severe hyperbilirubinemia when phototherapy is ineffective [20]. Peripheral arteriovenous exchange transfusion therapy which can ensure fluid balance and is easy to operate with a high safety factor, has been widely used in exchange transfusion for neonates [21]. This therapy needs establish three venous pathways for transfusion of red blood cells, plasma and liquid medicine respectively, and one arterial pathway for the discharge of blood. However, this arterial pathway was often blocked during the process of exchange transfusion, and the incidence could be up to 13.33-33.33%. Once arterial pathway is blocked, re-puncture and catheterization are required which prolong the time of blood exchange and increase the risk of infection. At present, heparin dilution solution is used for local anticoagulation through continuously micropump at the bleeding port of the artery [22]. But the dosage of heparin has not been clearly specified. The aim of this study is to investigate the effects of different doses of heparin on arterial occlusion rate and coagulation function in peripheral arteriovenous synchronous blood exchange transfusion therapy on neonatal hyperbilirubinemia, and to provide evidence about the optimal heparin dosage for clinical application. 

There were no significant differences in total bilirubin exchange rate, indirect bilirubin exchange rate, and hemoglobin value before and after exchange transfusion between the four groups (P > 0.05), which indicated that adding different dosages of heparin did not affect the efficacy of exchange transfusion treatment. In addition, there was no significant difference in arterial puncture points among the four groups (P > 0.05), indicating that the data of four groups were comparable. Significant difference in total rate of vascular occlusion was found between the four groups (P< 0.05), which were 15.6%, 31.9%, 21.3% and 8.7%, respectively. Further comparison showed that the rate of vascular occlusion of group A was significantly lower than that of groups B and C (P< 0.05). Through analyze, the usage of three-way infusion connector in groups B and C, which would induce the anticoagulant dead space and the occurrence of arterial blockage, may be the reason for the result mentioned above. The total vascular occlusion rate of group D (300 U/h) was the lowest, but there was no significant difference compared with group A (P > 0.05), suggesting that exchange transfusion treatment without heparinization in is safe and feasible in clinical practice, and could also reduce the treatment procedures and costs.

The axillary artery of neonates has a large diameter and less range of motion, but it is difficult to perform puncture; the brachial artery is deep in the body location and lacks collateral circulation, and the nerve usually is damaged during the puncture process; radial artery is the preferred site for neonatal arterial puncture catheterization due to its low difficulty and few complications [23]. However, the radial artery has the highest range of motion and difficulty in restricting during neonatal exchange transfusion. The blood flow at the radial end is frequently insufficient due to the wrist motion of the neonates. And if not handled in a timely manner, it may lead to blockage. Therefore, we further analyzed whether there was a difference between four groups in the blocking rate of different puncture sites. A significant difference in the rate of radial artery occlusion was found among the four groups (P < 0.05), the rate of radial artery occlusion in group B were highest (31.9%), while that in group D was the lowest (6.5%). The rate of radial artery occlusion between group D and group A was significantly different (P < 0.05). However, there was no significant difference in the rate of brachial artery occlusion and axillary artery occlusion among the four groups (P > 0.05). These results suggested that 300 U/h heparin could reduce the occurrence of occlusion when the puncture site of the exchange transfusion is radial artery, but heparin will be of no effect when the puncture site is the brachial or axillary artery.

Studies have shown that heparin has strong anticoagulant function through regulating multiple signaling pathways associated with the blood coagulation [24] [25]. Our results showed that the local anticoagulant effect was enhanced with the increase of dose heparin pumping to the bleeding artery, but the platelet number and APTT value showed no significantly difference between four groups before and after exchange transfusion (P > 0.05). These findings suggested that no heparin or different doses of heparin have little effect on coagulation function for the whole body of children, which may be related to the fact that a large amount of heparin pumped at the bleeding end of the artery is excreted with waste blood and only a small amount of heparin enters the body.

## Conclusion

In conclusion, the dose of heparin using in peripheral arteriovenous synchronous blood exchange transfusion on neonatal hyperbilirubinemia is affected by the site of arterial puncture. When the puncture site is the brachial or axillary artery, exchange transfusion without heparin may take precedence; but when the puncture site is radial artery exchange transfusion with 300 U/h heparin may be a better choice. Limited by time and sample size, whether it will affect the coagulation function of children with the increase of heparin dose needs to be further verified.

## Dodatak

### Funding

This study was supported by the Ganzhou City guiding science and technology project (GZ2018ZSF145).

### Conflict of interest statement

All the authors declare that they have no conflict of interest in this work.
